# Comparison of 1.5T and 3T MRI scanners in evaluation of acute bone stress in the foot

**DOI:** 10.1186/1471-2474-12-128

**Published:** 2011-06-06

**Authors:** Markus J Sormaala, Juha-Petri Ruohola, Ville M Mattila, Seppo K Koskinen, Harri K Pihlajamäki

**Affiliations:** 1 Research Department, Centre for Military Medicine, P.O. Box 50, FIN-00301 Helsinki, Finland; 2 Department of Orthopedic Surgery and Trauma, Tampere University Hospital, Tampere, Finland; 3 Department of Radiology, Helsinki University Central Hospital, Helsinki, Finland

## Abstract

**Background:**

Bone stress injuries are common in athletes and military recruits. Only a minority of bone stress changes are available on plain radiographs. Acute bone stress is often visible on MRI as bone marrow edema, which is also seen in many other disease processes such as malignancies, inflammatory conditions and infections. The purpose of this study was to investigate the ability of radiographs, 1.5T and 3T MRI to identify acute bone marrow changes in the foot.

**Methods:**

Ten patients with 12 stress fractures seen on plain radiographs underwent MRI using 1.5T and 3T scanners. T1 FSE and STIR axial, sagittal, and coronal view sequences were obtained. Two musculoskeletal radiologists interpreted the images independently and by consensus in case of disagreement.

**Results:**

Of the 63 acute bone stress changes seen on 3T images, 61 were also seen on 1.5T images. The sensitivity of 1.5T MRI was 97% (95% CI: 89%-99%) compared with 3T. The 3T MRI images where, therefore, at least equally sensitive to 1.5T scanners in detection of bone marrow edema. On T1-weighted sequences, 3T images were slightly superior to 1.5T images in visualizing the demarcation of the edema and bone trabeculae. The kappa-value for inter-observer variability was 0.86 in the MRI indicating substantial interobserver agreement.

**Conclusions:**

Owing to slightly better resolution of 3T images, edema characterization is easier, which might aid in the differential diagnosis of the bone marrow edema. There was, however, no noteworthy difference in the sensitivity of the 1.5T and 3T images to bone marrow edema. Routine identification of acute bone stress changes and suspected stress injuries can, therefore, be made with 1.5T field strength.

## Background

MRI plays an important roll in the diagnosis of stress fractures and in imaging acute bone stress changes in bone [1]. In the foot, MRI offers an accurate means of identifying acute bone stress changes in the small bones [2]. Recent advances in 3T MRI systems offer significant advantages for musculoskeletal imaging [3-5]. The better signal-to-noise ratio can be utilized in imaging ligaments and cartilages as well as meniscal structures of the knee [6-8]. Some previous studies of the knee [9-11] indicate the 3T images have excellent sensitivity and specificity for detecting meniscal tears and ACL ruptures compared to arthroscopy.

In the foot, high quality 3T MRI images enable accurate diagnosis of collateral ligament and syndesmosis injuries [12]. The foot is a suitable subject for 3T MRI as it is not susceptible to many of the problems involved with 3T imaging. It is easy to position in the isocenter of the magnetic field where the field is most homogenous. The patient can also be placed in a comfortable position and it is not close to the heart or lungs, therefore is not prone to movement during imaging. The 3T images should also be superior in imaging small periarticular erosions and erosions in the cartilages of the small joints of the foot [12]. There is, however, only a limited amount of studies on the use of 3T MRI on the foot. So far, the published studies have focused on characterizing the anatomy of the foot [13] and on developing parallel imaging [14]. To the authors' knowledge, no studies have yet been published describing the accuracy of 3T images in the evaluation of the bone stress changes of the foot.

The purpose of the present diagnostic study was to compare sensitivity of plain radiographs, 1.5T and 3T MRI in the diagnostic evaluation of acute bone marrow changes and stress fractures in the foot and ankle.

## Methods

A total of 10 patients were recruited from military garrisons over a six-month period. All the patients enrolled into the study were performing their compulsory military service. In the authors' country, all male citizens are obliged to perform a six, nine, or 12-month-long military service, whereas women may volunteer for the service. Annually, an average of 23,000 men and 370 women complete the service. Of any specific age group, over 80% of men enter the service, most before turning 21 years old (median age, 19 years) [2,15].

**Figure 1:**
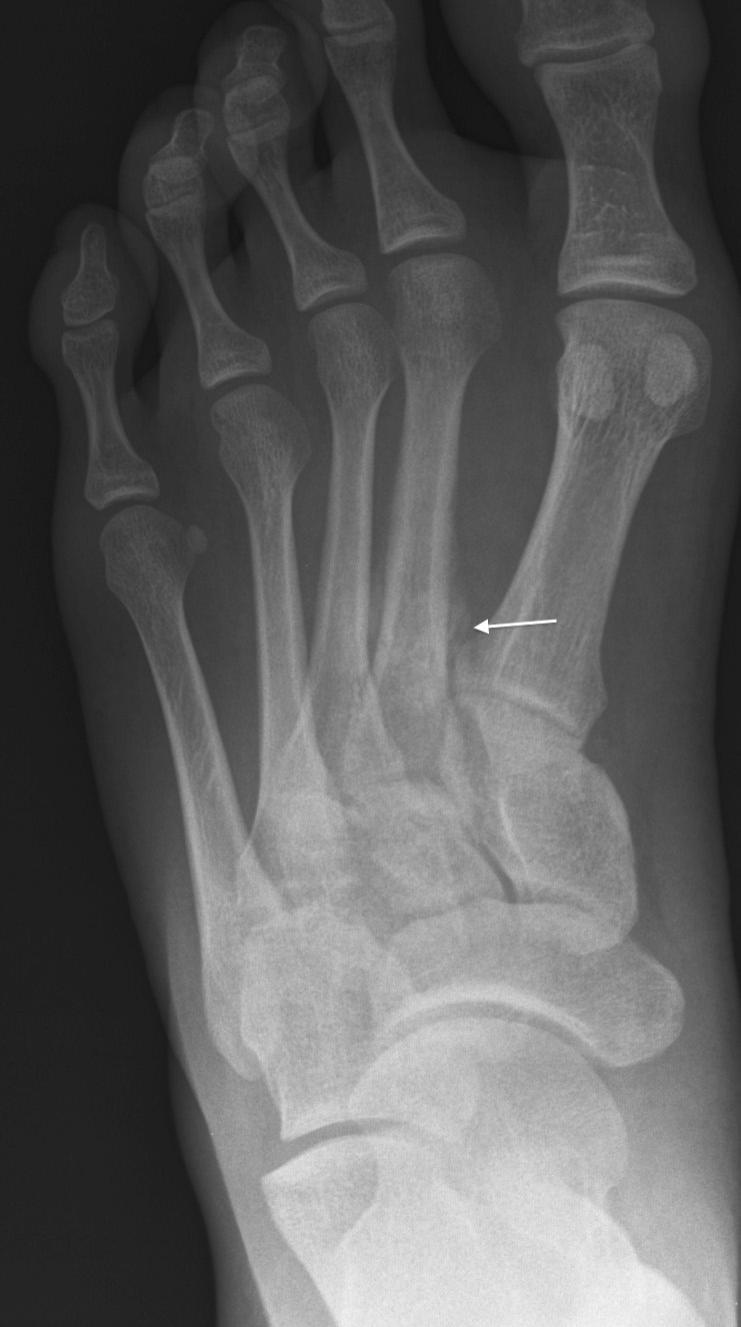
**Plain film radiograph of 20-year-old patient with foot pain associated with exercise**. Subtle periostial reaction indicated by arrow at base of second metatarsal bone indicates stress fracture.

The inclusion criteria for the study were pain during exercise in the ankle or foot, and a fracture line, callus or faded cortex on plain radiography indicating a stress injury of the foot. Antero-posterior and oblique views of the foot where obtained. The radiographic finding of the patients included periostial reactions, fracture lines or sclerosis (Figure 1). Patients with recent minor or older major trauma were excluded from the study. Also patients with infections or suspected malignancies were excluded. The medical ethics committee of the hospital district of Helsinki and Uusimaa approved the study design. Written informed consent was obtained from all the patients for participation in the study and publication of accompanying images.

The patients were referred from the garrisons to a single hospital, where they underwent imaging with both a Siemens MAGNETOM Symphony 1.5-T magnet and a Siemens MAGNETOM Trio 3T magnet (Siemens Medical Solutions Inc). The patients were imaged with the two scanners within four hours. T1 SE and STIR sagittal view, T1 SE and STIR axial view, and T1 SE and STIR coronal view sequences were obtained with both scanners. The exact imaging parameters are presented in Table 1. Both scanners were equipped with chimney type extremity coils.

**Table 1:** Imaging parameters used in the study. Slightly different TR and TE values were used for different planes

	3T		1.5T	
	**STIR**	**T1**	**STIR**	**T1**

TR	6780-8330	504-620	4450-6780	450-652

TE	78-68	12-16	67-68	12-13

TI	180	-	130	-

NEX	1	1	1	1

Slice Thickness	3	3,5	3	3

Gap	0.6	0.55	0.6	0.6

Matrix (axial)	448X358	384X307	254X173	448X224

Pixel BW	150	160	145	130

ETL	15	1	7	1

STIR = Short Tau Inversion Recovery, TR = repetition time, TE = time to echo, TI = inversion time, NEX = number of excitations, BW = Bandwidth, ETL = Echo Train Length

Two musculoskeletal radiologists with six and 22 years of experience reviewed images first separately and, in case of disagreement, together to reach consensus. The kappa-value for inter-observer variability was 1.0 in the plain film radiographs and 0.86 in the MRI indicating substantial inter observer agreement. Every study was evaluated independently with the purpose of determining with the best possible accuracy the extent of the acute bone stress changes and bone stress injury in the foot or the ankle. The specific diagnostic signs focused on were: 1) how well the edema was visualized, 2) how well the borders of the edema demarcated, and 3) how well the bony trabeculae of spongious bone were visualized. The diagnostic quality of each sign on the 3T images was subjectively classified as superior to, inferior to, or equal to that of the corresponding sign on the 1.5T image. The acute bone stress changes and stress injuries found where graded as follows; grade I endosteal marrow edema, grade II periosteal and endosteal edema, grade III muscle, periostial and endosteal edema, grade IV fracture line, grade V fracture line with callus [16]. The total results of 3T and 1.5T images were compared to evaluate the possible discrepancy in the bone marrow edema of the bone stress injuries between the two studies. In addition, the osteochondral structures and ligaments of the foot and the ankle were assessed.

## Results

During the study period 10 male patients (age range, 19-20 years; median age, 19 years) enrolled and were identified with single or multiple stress injuries in 11 feet. All the patients enrolled in the study had pain in the foot during exercise and generalised tenderness on physical exam. Detailed information about the distribution and grade of the stress injuries are shown in Table 2. All the patients had served in the military for four months or less. Acute bone marrow changes such as edema were seen in 63 bones, 13 of these were stress fractures with a low-signal-intensity fracture line identifiable on MRI. In 51 bones only acute bone marrow changes, without visible fracture lines, were seen. Twelve of the 13 stress fracture grade stress injuries were seen in the metatarsal bones and one in the cuboideum. All of the stress fractures seen in the metatarsal bones could be seen on the radiographs. In addition to these 12 metatarsal fractures, one additional fracture in the cuboideum was detected with MRI. The number of incidences acute stress changes in a single foot ranged from one to 11 with an average of five affected bones per foot. The lower grade stress changes were seen in the metatarsal bones, cuboideum, all cuneiforms, naviculare, talus, calcaneus, tibia, and fibula. No other pathology than the stress acute injuries and acute bone marrow changes were seen in any of the patients.

**Table 2:** Distribution and grade of bone stress changes and stress injuries in study patients

Patient	Age	Side	Scanner	TIB	FIB	TAL	CAL	NAV	CUB	CM	CI	CL	MT1	MT2	MT3	MT4	MT5	Total
P1	19	R	1,5T					I		I	I	I		V	II			6

P1			3T					I		I	I	I		V	II			6

P1			Radiograph											V				1

P2	20	L	1,5T			I		I	I	I		I	V	I	I	I		9

P2			3T			I		I	I	I		I	V	I	I	I		9

P2			Radiograph										V					1

P3	19	R	1,5T					I	I	I	I	I	I		V			7

P3			3T					I	I	I	I	I	I		V			7

P3			Radiograph												V			1

P4	19	R	1,5T			I	I	I	I	I	I	I	IV	I				9

P4			3T			I	I	I	I	I	I	I	IV	I				9

P4			Radiograph										IV					1

P4		L	1,5T			I	I	I	I	IV	I	I	IV	II				9

P4			3T			I	I	I	I	IV	I	I	IV	II	I		I	11

P4			Radiograph										IV					1

P5	19	L	1,5T											V				1

P5			3T											V				1

P5			Radiograph											V				1

P6	19	R	1,5T											V				1

P6			3T											V				1

P6			Radiograph											V				1

P7	19	L	1,5T											V				1

P7			3T											V				1

P7			Radiograph											V				1

P8	20	L	1,5T			I	I	I		I					V	V		6

P8			3T			I	I	I		I					V	V		6

P8			Radiograph												V	V		1

P9	20	L	1,5T			I					I			I	V			4

P9			3T			I					I			I	V			4

P9			Radiograph												V			1

P10	19	L	1,5T					I	I	I	I	I	I		I	V		8

P10			3T					I	I	I	I	I	I		I	V		8

P10			Radiograph													V		1

Number in column indicates grade of stress injury. Last column indicates total number of stress injuries in the patient. The 3T images found two low grade stress injuries in the left foot of patient 4 which did not visualize in the 1.5 T images. (TIB = tibia, FIB = fibula, TAL = talus, CAL = calcaneus, NAV = naviculare, CUB = cuboideum, CM = medial cuneiforme, CI = intermediale cuneifrome, CL = lateral cuneiforme, MT1 = first metatarsal, MT2 = second metatarsal, MT3 = third metatarsal, MT4 = fourth metatarsa, MT5 = fifth metatarsal)

**Figure 2:**
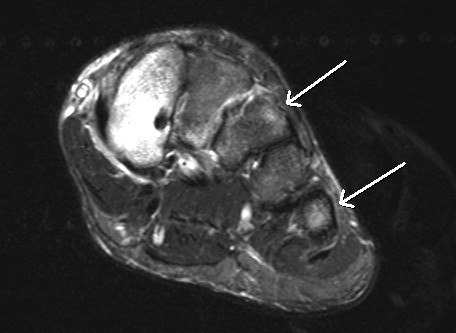
**Axial STIR 3T images of a 19-year-old patient with bone stress injuries in the metatarsal bones**. Subtle bone marrow edema can be seen on the third and fifth metatarsal bones (arrows).

**Figure 3:**
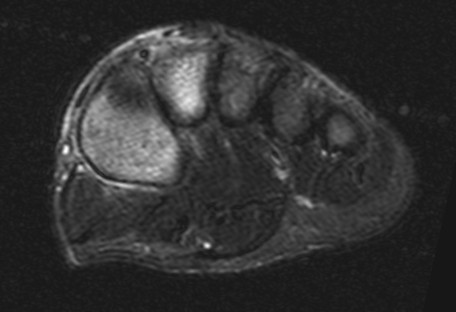
**Axial STIR 1.5T images of the same patient as in the 3T images of figure 2**. The subtle bone marrow edema seen in the 3T images cannot reliably be seen in these 1.5T images.

**Figure 4:**
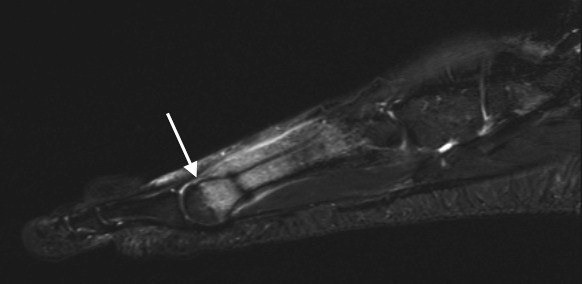
**Sagittal STIR 3T images of a 19-year-old patient with a stress fracture in the left IV metatarsal bone**. Bone marrow edema at the epiphysis (arrows) demarcates more clearly in figure 4, 3T image compared to figure 5, 1.5T image.

**Figure 5:**
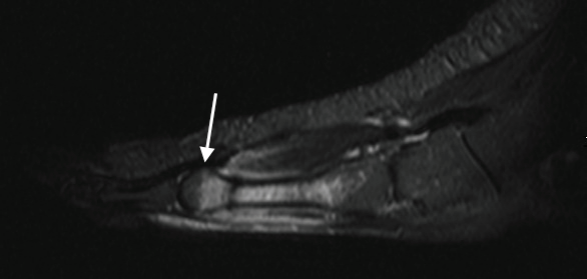
**Sagittal STIR 1.5T images of the same patient as in the 3T images of figure 4**. Bone marrow edema at the epiphysis (arrows) demarcates more clearly in figure 4, 3T image compared to figure 5, 1.5T image.

Twelve bone stress injuries with fracture lines were visible on plain radiographs. The sensitivity of plain radiographs was thus 92% (95% CI: 65-100%) and positive predictive value 100% (95% CI: 76-100%) compared with 3T scanner in detecting bone stress injuries with fracture lines. The 1.5T MRI scanner successfully identified 61 out of the 63 visible incidences of acute bone marrow detected on the 3T MRI scans (sensitivity 97%; 95% CI: 89% to 99%) (Figure 2 & Figure 3). There was therefore no noteworthy difference in the sensitivity of the 1.5T and 3T images in the sensitivity to bone marrow edema. The edema signal seen on the 3T images was, however, also more homogenous, and its boundaries could be defined more accurately. Especially where the edema was limited to the epiphyseal lines, the demarcated borders of the edema showed more clearly on the 3T images (Figure 4 & Figure 5).

**Figure 6:**
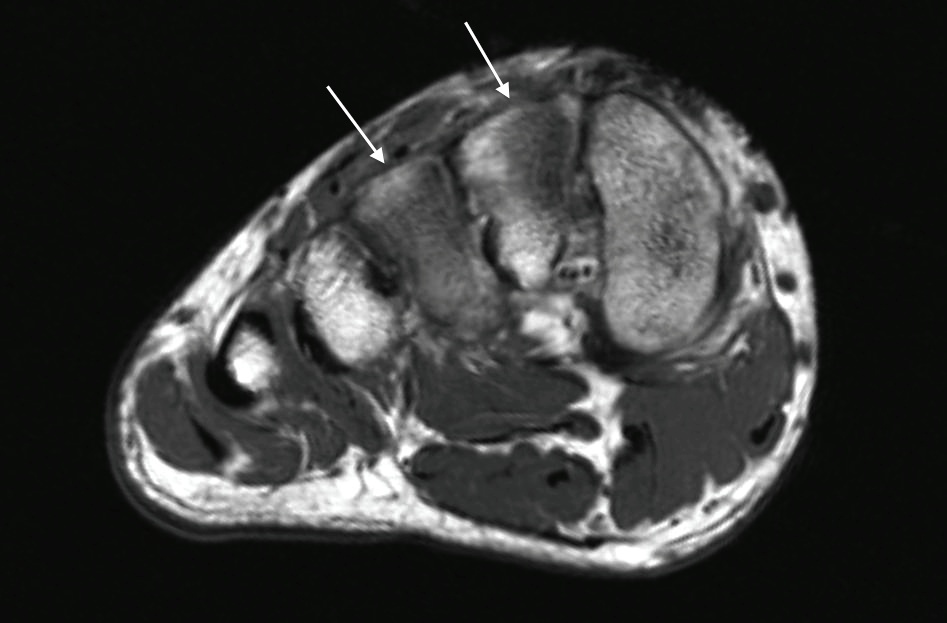
**Axial T1 3T images of a 19-year-old patient with a stress fracture in the right III metatarsal bone**. The bone trabeculae (arrows) visualize better in figure 6, the 3T images compared to figure 7, the 1.5T images.

**Figure 7:**
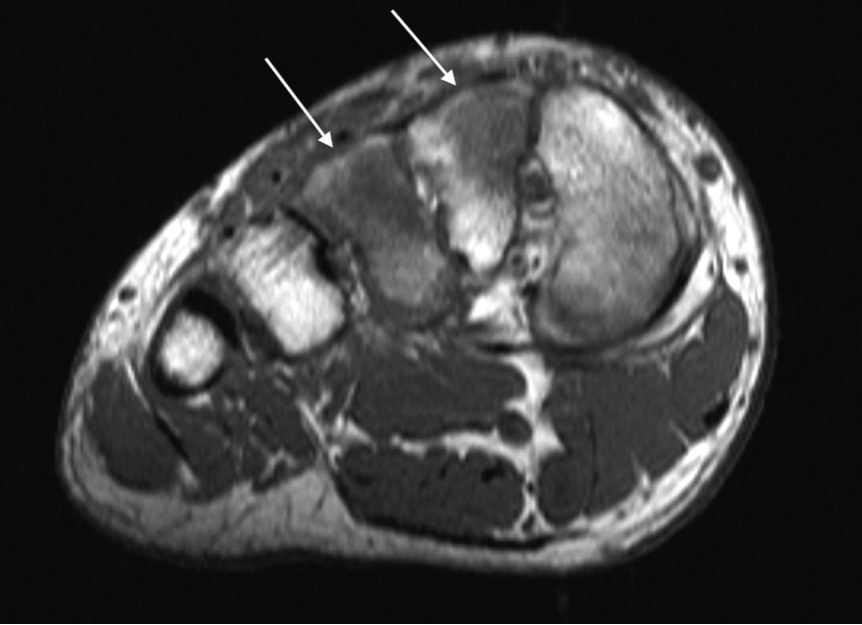
**Axial T1 1.5T images of the same patient as in the 3T images of figure 6**. The bone trabeculae (arrows) visualize better in figure 6, the 3T images compared to figure 7, the 1.5T images.

On the T1-weighted images with FSE sequence, however, the bone marrow edema was more easily visible at the 3T field strength compared with the 1.5T. Another difference between the T1-weighted 3T and 1.5T images was that the better resolution achieved by the 3T field strength allowed visualization of the trabecular structures of spongious bone. The bone trabeculae could, consequently, be evaluated from the 3T images but not from the 1.5 T images (Figure 6 & Figure 7).

## Discussion

Bone stress changes of the foot are generally considered benign and self-limiting with reduced exercise. When symptomatic they can, however, cause significant problems for athletes trying to focus on their training program, or for military recruits who might even be forced to interrupt the service due to severe, recurring stress injuries in the feet [17,18]. Making a swift and accurate diagnosis in these symptomatic patients is essential to ensure appropriate treatment. According to our study, 3T higher field strength MRI scanners are equally sensitive to equal 1.5T scanners MRI scanners in detecting bone stress edema.

The distinct feature of bone stress changes in the foot is that, in most cases, they occur in several bones simultaneously. For example, in our study, the patients had an average of five affected bones in their feet. Stress fractures are seen on plain film radiographs, whereas MRI also shows the acute bone stress changes visible only as edema in the marrow and surrounding periostium [2]. In our study the MR-images, however, also showed one additional stress fracture in the medial cuneiform of one of the patients, which was not seen on the radiographs. Based on our results, 3T MRI can be considered a good tool in evaluating the extent of acute bone marrow changes in the foot and enables the correlation these finding with the clinical status of the patient. Both 3T and 1,5T images provide the clinician with a good estimate of the extent of acute bone stress changes in the foot.

Imaging of bone marrow edema is one of the fundamental features of musculoskeletal MRI. Edema can be seen in association with trauma, infections, and malignancy. Although in many cases, the etiology of the edema is obvious, it can also sometimes present a differential diagnostic challenge. Our study demonstrated that the 3T MR imaging systems are at least as good as or in some cases slightly better to the 1.5T systems for imaging and characterizing bone marrow edema. Even though the edema in all our patients was due to acute bone stress changes, our results are generalizable to all types of bone marrow edema. Acute bone stress changes can be used as a good model for all types of edema.

The better visualization of bone trabeculae in T1-weighted images and the clearer demarcation of the edema in the STIR images might be useful in the differential diagnostics of the edema. High-resolution 3T MRI images may in future prove to have advantages over 1.5T in diagnosis of conditions characterised by trabecular destruction, such as malignancy and infection. This might help differentiate the edema seen in trauma from the edema in malignancies and infections. Characterizing the T1 bone marrow signal has been proven to be important in evaluating osteomyelitis [19]. The most important finding of our study was the improved characterisation of both the edema and the bone trabeculae on 3T images as compared to 1.5T images. Therefore, we might expect that 3T images would be more useful in evaluating osteomyelitis. Also the limiting of the edema into the epiphyseal lines is another way to characterize it and might help in the differential diagnosis. It must, however, also be considered, that 3T scanners also have potential disadvantages compared to 1.5T scanners. 3T MRI is currently not widely available and costs of scanning are sometimes higher. Also some artifacts might be more prominent in 3T images compared to 1.5T images.

Although all participants in our study were symptomatic due to their acute stress fractures, it remains unclear as to the importance and natural history of the many incidences of non-fracture acute bone stress changes observed in this sample of a high risk population. These acute bone stress changes were used in the study only as a model of bone marrow edema. Prospective MRI studies of initially asymptomatic army recruits may provide valuable insights in the development, prevention and management of bone stress injuries in exercising populations.

## Conclusions

Our study shows that 3T images are generally at least equal to 1.5T images for the diagnosis of bone stress changes. Based on our results, 1.5T images can be considered adequate in routine stress fracture diagnosis, as even low-field MRI has been proven sufficient in the diagnosis of lower extremity pain [20,21]. However, in future 3T images may also contribute to the evaluation of other conditions affecting bone such as infection and malignancy.

## Competing interests

The authors declare that they have no competing interests.

## Authors' contributions

All authors participated in the study design and to the planning of the project. All authors also closely co-operated in analyzing the results and preparing the manuscript. MJS drafted and submitted the final manuscript. J-PR recruited a majority of the patients in the study. VMM performed the statistical analysis. MJS and SKK performed the image reading. HKK coordinated the management and administration of the project. All authors read and approved the manuscript before submission.

## Pre-publication history

The pre-publication history for this paper can be accessed here:

http://www.biomedcentral.com/1471-2474/12/128/prepub
